# Integrated OMICs approach reveals energy metabolism pathway is vital for *Salmonella* Pullorum survival within the egg white

**DOI:** 10.1128/msphere.00362-24

**Published:** 2024-06-11

**Authors:** Xiamei Kang, Hongli An, Baikui Wang, Linlin Huang, Chenghu Huang, Yingying Huang, Zining Wang, Fang He, Yan Li, Min Yue

**Affiliations:** 1 Department of Veterinary Medicine, Zhejiang University College of Animal Sciences, Hangzhou, China; 2 Hainan Institute of Zhejiang University, Sanya, China; 3 State Key Laboratory for Diagnosis and Treatment of Infectious Diseases, National Clinical Research Center for Infectious Diseases, National Medical Center for Infectious Diseases, The First Affiliated Hospital, College of Medicine, Zhejiang University,, Hangzhou, China; Martin Luther University of Halle-Wittenberg Institute of Biology/Microbiology, Halle (Saale), Germany

**Keywords:** *Salmonella *Pullorum, egg white, survival strategy, RNA-Seq, comparative genomic analysis

## Abstract

Eggs, an important part of a healthy daily diet, can protect chicken embryo development due to the shell barrier and various antibacterial components within the egg white. Our previous study demonstrated that *Salmonella* Pullorum, highly adapted to chickens, can survive in the egg white and, therefore, be passed to newly hatched chicks. However, the survival strategy of *Salmonella* Pullorum in antibacterial conditions remains unknown. The overall transcripts in the egg white showed a large-scale shift compared to LB broth. The expression of common response genes and pathways, such as those involved in iron uptake, biotin biosynthesis, and virulence, was significantly changed, consistent with the other transovarial transmission serovar Enteritidis. Notably, membrane stress response, amino acid metabolism, and carbohydrate metabolism were specifically affected. Additional upregulated functionally relevant genes (JI728_13095, JI728_13100, JI728_17960, JI728_10085, JI728_15605, and *nhaA*) as mutants confirmed the susceptible phenotype. Furthermore, *fim* deletion resulted in an increased survival capacity in the egg white, consistent with the downregulated expression. The second-round RNA-Seq analysis of the Δ*fim* mutant in the egg white revealed significantly upregulated genes compared with the wild type in the egg white responsible for energy metabolism located on the *hyc* and *hyp* operons regulated by FhlA, indicating the Δ*fim* mutant cannot receive enough oxygen and switched to fermentative growth due to its inability to attach to the albumen surface. Together, this study provides a first estimate of the global transcriptional response of *Salmonella* Pullorum under antibacterial egg white and highlights the new potential role of *fim* deletion in optimizing energy metabolism pathways that may assist vertical transmission.

**IMPORTANCE:**

Pullorum disease, causing serious embryo death and chick mortality, results in substantial economic losses worldwide due to transovarial transmission. Egg-borne outbreaks are frequently reported in many countries. The present study has filled the knowledge gap regarding how the specific chicken-adapted pathogen *Salmonella* Pullorum behaves within the challenging environment of egg white. The deletion of the *fim* fimbrial system can increase survival in the albumen, possibly by reprogramming metabolism-related gene products, which reveals a new adaptive strategy of pathogens. Moreover, the comparison, including previous research on *Salmonella* Enteritidis, capable of vertical transmission, aims to provide diversified data sets in the field and further help to implement reasonable and effective measures to improve both food safety and animal health.

## INTRODUCTION

Eggs are of high nutritional value, representing one of the important components in a healthy daily diet (1). Egg white, also called albumen, constitutes ~70% of the total weight of an egg, primarily composed of water (~88%) and proteins (~10.5%), with a small amount of carbohydrates (~0.5%) (2). More than 150 proteins were identified in the albumen using proteomics and mass spectrometry (3). Among these proteins, many exhibit antibacterial activity (4). Ovotransferrin, accounting for about 12% of egg white protein, is a high-affinity iron-chelating protein that restricts bacterial replication by competing for iron. Ovomucoid, one of the protease inhibitors, hinders bacterial proteases required to colonize the host. Lysozyme lyses the cell wall of most bacteria, resulting in membrane damage. Additional vitamin-binding proteins like avidin and flavoprotein, which sequester biotin and flavoprotein, respectively, limit bacterial respiration and metabolism (4). Certain small proteins with DNase activity can cause DNA damage to bacteria (5). Finally, the alkaline pH in egg white can enhance cell membrane permeability and disrupt pH homeostasis, which has been proven to be an antimicrobial mechanism (6).

However, specific bacterial pathogens like *Salmonella* Pullorum (*S*. Pullorum) and *Salmonella* Enteritidis (*S*. Enteritidis) can survive in antibacterial egg white and are persistently carried in chicken embryos and chicks (7–9), which is an essential step in transovarial transmission. We speculate that these bacteria with vertical transmission capabilities probably employ a unique survival strategy to persist in the hostile condition of egg albumen. So far, several studies have explored the survival mechanisms of *S*. Enteritidis due to its significance as a common zoonotic *Salmonella* serovar frequently associated with ingesting eggs and egg products (8). Various approaches, including targeted gene mutagenesis (10), random mutagenesis (11), *in vivo* expression technology (12), and transcriptional analysis (13), were employed to understand the genetic determinants of the survival strategy. Furthermore, some experimental conditions, i.e., temperature and pH, have been proven to play a critical role in antimicrobial activity (10, 14). For example, at a higher temperature like 45°C, egg white contributes minimal antibacterial effects against *S*. Enteritidis (15). Nevertheless, there is a huge knowledge gap on the survival strategies of *S*. Pullorum in egg white.

Generally, most studies about *S*. Enteritidis provide important information for understanding the survival of *S*. Pullorum in antibacterial egg white, considering these two agents have a close genetic relationship (16). However, even among two *S*. Enteritidis strains with similar genetic backgrounds, their survival capabilities in egg white show considerable differences under the identical examined condition (17). Moreover, *S*. Pullorum is a host-adapted pathogen with only six fimbrial appendages (18), whereas *S*. Enteritidis is a serovar with a broad host range and 13 fimbrial gene clusters (19). There are more pseudogenes within the *S*. Pullorum (16, 18, 19). Furthermore, Pullorum disease, caused by *S*. Pullorum, is one of the leading causes of embryonic and young chick death worldwide, with a hallmark vertical transmission capability. In China, the prevalence rate within hens is 13.88%, approximately twice that observed in cocks (20), indicating a significant role in transovarial transmission. The evidence that *S*. Pullorum-infected chickens exhibited ovarian lesions and produced contaminated eggs can be traced back to 1914 (21–23). Moreover, *S*. Pullorum could be detected in the ovaries and oviducts of infected hens, with higher bacterial loads observed in the oviduct (24, 25), through which eggs develop into the yolk and albumen, respectively. The detection rate of *S*. Pullorum from the albumen of eggs laid by experimentally infected hens reached as high as 35% (26). The above facts suggest that investigating mechanisms employed by *S*. Pullorum to withstand the challenging egg white environment is imperative, which could guide novel interventions that might break down the transovarial transmission.

Here, transcriptional sequencing analysis was used to obtain a global gene-expression profile of *S*. Pullorum exposed to egg white at 42°C, mimicking the condition during egg formation. Using integrated RNA-seq and comparative genomic analysis, five functionally related genes were selected to construct deletion mutants, with their survival ability in egg albumen significantly lower than that of the wild-type strain, highlighting their crucial role in survival in egg albumen. We also looked into unique downregulated genes specific for Pullorum persistence and found that the absence of the *fim* gene cluster notably improved proliferation in egg white, possibly by reprogramming gene products related to energy metabolism, shedding new insights into survival strategies by *S*. Pullorum.

## RESULTS

### The behavior of *S*. Pullorum within the egg albumen

**Fig 1:**
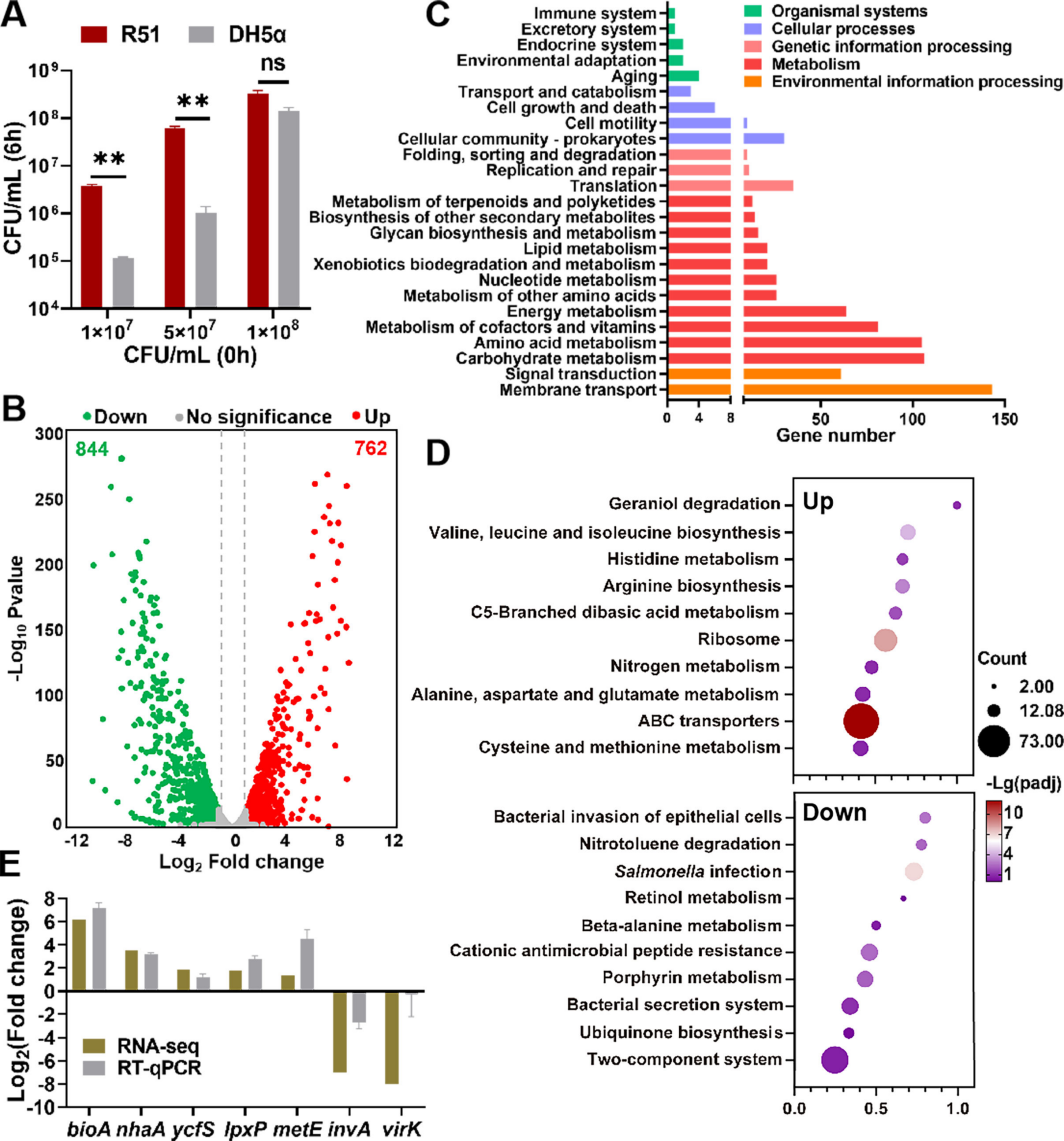
The behavior of *S*. Pullorum within the egg albumen. (**A**) The selection of bacterial inoculum concentration. (**B**) Volcano plot of the gene expression. The red dots represent the upregulated differentially expressed genes (DEGs), and the green dots represent the downregulated DEGs. (**C**) KEGG pathway classifications of DEGs. (**D**) The upregulated and downregulated KEGG pathways’ enrichment analysis for the top 10 terms with smaller adjusted *P*-values. Each dot represents a pathway. The rich factor indicates the proportion of DEGs in the pathway. The circle size reflects the number of DEGs, and the color represents the range of the *P*-value. (**E**) The expression of selected genes was validated by RT-qPCR. Comparisons between RNA-seq and qPCR data with RNA samples exposed to albumen for 6 h. The relative expression level of target genes was measured with the 2^–ΔΔCt^ method. The unpaired *t*-test was used to analyze the differences between R51 and DH5α. *P < 0.05; **P < 0.01.

Since high bacterial concentrations could hinder the antibacterial activity of egg albumen due to inadequate local antibacterial components (10, 27), we examined the viability of two strains, R51 and DH5α, at three different concentrations to maximize the bacterial quantity for transcriptomic sequencing. The *Escherichia coli* (*E. coli*) strain DH5α was selected as a control due to its relatively weak survival capability in egg white (11). When the inoculum doses were 1 × 10^7^ and 5 × 10^7^ CFU/mL, R51 showed a significantly higher count than DH5α after 6 h. However, once it reached 1 × 10^8^ CFU/mL, egg white no longer inhibited the growth of DH5α, and no difference was observed between the two strains (Fig. 1A). Therefore, we prepared the RNA-Seq samples using an initial inoculum concentration of 5 × 10^7^ CFU/mL.

After exposure to egg albumen and Luria-Bertani (LB) broth at 42°C for 6 h at the same inoculum size, the cultures were collected to extract the total mRNA for RNA sequencing to provide a global pattern of gene expression. The results demonstrated that more than 99% of clean reads were mapped to the genomic sequence after removing ribosomal RNA (rRNA). A total of 4,260 genes were transcribed in both culture media. Among these, 1,606 genes were differentially expressed [false discovery rate, FDR ≤ 0.05 and |log_2_(fold change) | ≥1; Table S1], accounting for about 37% of the whole genome, which suggested a substantial alteration of the transcriptional response in the presence of egg white. Compared to the LB broth, there were 762 genes differentially upregulated in the egg albumen, while 844 genes were significantly downregulated (Fig. 1B).

Among the differentially expressed genes (DEGs), we observed significant stimulation of iron-starvation genes (Table S2), indicating limited iron availability in egg white, leading *Salmonella* to produce ferric chelators called siderophores (28, 29). Moreover, the genes *mntH* and *sitABCD* related to manganese transport systems were significantly upregulated, suggesting a strong induction in response to the low manganese (28, 30). Additionally, genes related to biotin synthesis were notably upregulated. Amino acid synthesis and transport genes and carbohydrate metabolism-related genes were strongly (Table S2) induced, highlighting amino acids and carbohydrates as key nutrients in albumen. Genes involved in aerobic and/or anaerobic respiration decreased upon albumen exposure, including hydrogenases (*hybADEF*, *hycCDEFGHI*, *hydN*, and *hypBC*), succinate dehydrogenase (*sdhCDAB*), and fumarate (*dmsABC*), consistent with the previous study (13). Virulence-related genes, including those in *Salmonella* pathogenicity island 1 (SPI-1) and SPI-2, were significantly downregulated, indicating reduced invasiveness in the adverse environment. Notably, all six fimbriae appendages in *S*. Pullorum showed downregulation, particularly the *fim* gene cluster exhibiting complete downregulation (Table S2).

To understand the pathways and gene interactions under albumen pressure, all the differentially expressed genes were subjected to pathway enrichment analysis based on the Kyoto Encyclopedia of Genes and Genomes (KEGG) database and assigned to over 20 pathways (Fig. 1C; Table S3). The most prominent KEGG enrichment pathways were membrane transport, carbohydrate metabolism, and amino acid metabolism, with 143 (87 upregulated and 56 downregulated), 106 (49 upregulated and 57 downregulated), and 105 (81 upregulated and 24 downregulated) genes enriched, respectively. The former belonged to environmental information processing, while the latter two were related to metabolism (Fig. 1C). Furthermore, KEGG pathway enrichment analysis of DEGs was conducted, and it showed that among the top 10 enriched pathways based on the adjusted *P*-value for upregulated genes, some specific amino acid metabolic and biosynthetic pathways were enriched in response to albumen stress, including histidine metabolism, valine-leucine-isoleucine biosynthesis, and arginine biosynthesis (Fig. 1D). Moreover, the majority of the enriched pathways in the downregulated DEGs were associated with *Salmonella* virulence factors, such as bacterial invasion of epithelial cells, the bacterial secretion system, and *Salmonella* infection (Fig. 1D).

To validate the expression profile observed by RNA-Seq, five upregulated and two downregulated DEGs were selected to perform real-time quantitative polymerase chain reaction (RT-qPCR) assays. Genes *bioA,* vital for biotin biosynthesis, and *nhaA,* encoding Na^+^/H^+^ antiporter (31), were significantly increased by RT-qPCR assay, which was also upregulated in egg albumen (log_2_ fold change = 6.20 and 3.51, respectively) (Fig. 1E), confirming the reliability of the RNA-Seq analysis.

### Integrated OMICs to screen functionally relevant genes

**Fig 2:**
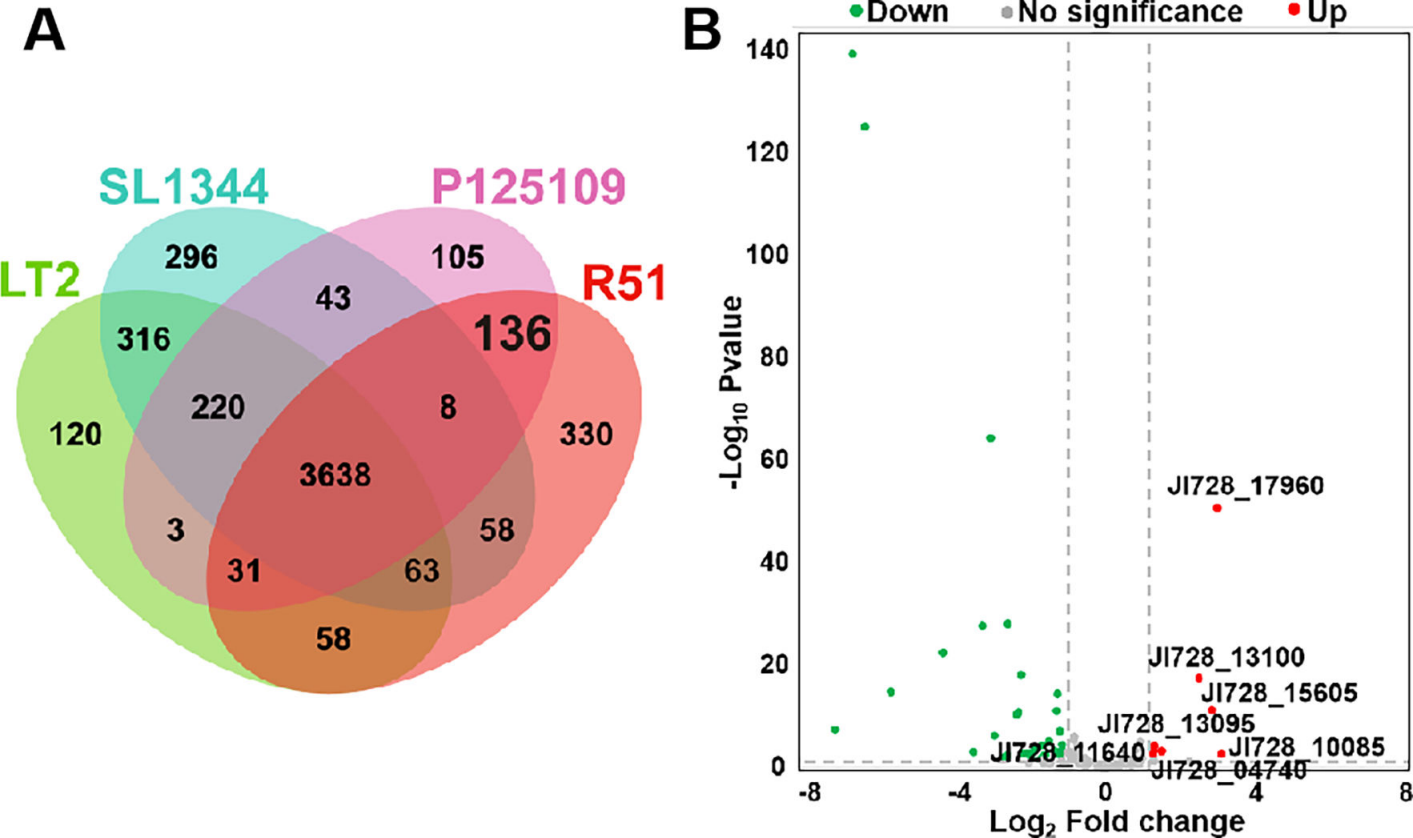
Integrated OMICs to screen functionally relevant genes. (**A**) Venn diagram showing the numbers of unique and shared genes among four strains. (**B**) Volcanic map of 136 genes exclusively shared by R51 and P125109. The red dots represent the upregulated DEGs, and the green dots represent the downregulated DEGs. The gene JI728_13100, in red, belongs to the carbohydrate metabolism pathway in the KEGG database.

*S.* Pullorum and *S*. Enteritidis are acknowledged pathogens capable of vertical transmission (7, 9, 23, 32, 33), whereas *Salmonella* Typhimurium could colonize the reproductive tract of hens but fails to colonize laid eggs, suggesting a lack of vertical transmission ability (5, 34). Specific factors, such as unique genes responsible for vertical transmission, are likely present in vertically transmissible bacteria. Based on this, by integrating transcriptomic data, we performed a comparative genomics method to identify crucial genes responsible for the survival of *S*. Pullorum in egg albumen. R51, one *S*. Enteritidis strain P125109, and two *S*. Typhimurium strains SL1344 and LT2, for which whole-genome sequencing has been completed, were selected for further analysis. A presence/absence variation matrix among the strains, including the core genes and strain-specific genes, was obtained with a web-based tool PanExplorer (Table S4), which was visualized as a Venn diagram (Fig. 2A) (35). There were 136 genes exclusively shared by *S*. Pullorum R51 and *S*. Enteritidis P125109, of which seven genes were differentially upregulated and 32 genes downregulated when mapped onto the transcriptomic data (Fig. 2B). Of note, one of the upregulated genes, JI728_13100, belongs to the carbohydrate metabolism in the KEGG database (Fig. 1C), which is associated with the enriched pathway pentose and glucuronate interconversions.

### Upregulated metabolic genes confirm survival fitness

**Fig 3:**
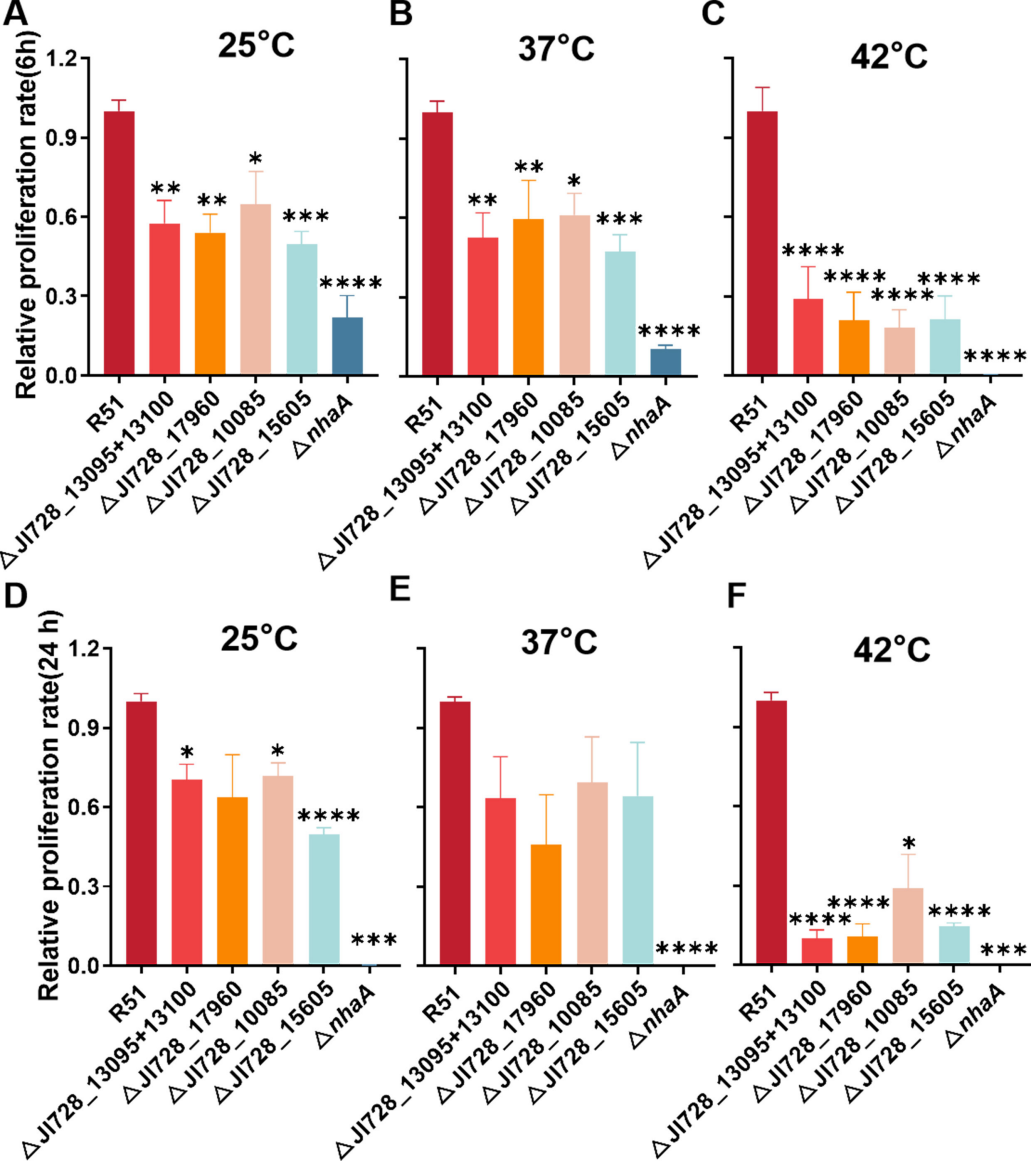
Upregulated metabolic genes confirm survival fitness. The wild-type strain R51 and its five derivatives were incubated in egg albumen at 25°C, 37°C, and 42°C for 6 h (**A–C**) and 24 h (**D–F**). The data were presented with the relative proliferative rate of mutants to the wild type. Mean values and SEM were displayed. The ANOVA test was used to analyze the differences between wild type and mutants. *P < 0.05; **P < 0.01; ***P < 0.001; and ****P < 0.0001.

To investigate the impact of the upregulated genes on the viability of *S*. Pullorum in egg white, five single gene deletion mutants were constructed using a previous method (18). Among them, JI728_13095 and JI728_13100 were considered as a whole for deletion due to their close genomic proximity and involvement in the metabolic process, while the other four were individually deleted (JI728_17960, encoding sel1 repeat family protein, JI728_15605 and JI728_10085, encoding uncharacterized protein, and *nhaA,* encoding Na^+^/H^+^ antiporter). The gene *nhaA* was chosen as a reference because it plays a crucial role in the survival of egg white (13). Additionally, the gene JI728_04740, encoding a potential cytotoxin protein, was not considered. Unfortunately, attempts to delete the gene JI728_11640 were unsuccessful, indicating a likely essential gene for the bacteria. The wild-type strain and mutants were inoculated in 80% (vol/vol) of egg albumen for 6 and 24 h at 25°C (storage temperature), 37°C (incubation), and 42°C (hatch), after which their survivability was determined. It was evident that the survival capability of mutants in egg white was significantly lower than that of the wild-type strain (Fig. 3A through F) (identical growth rates in LB broth, data not shown). However, survival conditions at 28°C and 37°C slightly improved after 24 h compared to 6 h, possibly due to the provision of nutrients by partially deceased bacteria (10). Moreover, the effects of these genes were temperature-dependent, with the most significant impact observed at 42°C. This was likely due to high temperatures causing bacterial membrane damage (6, 36), requiring the involvement of genes related to metabolism (JI728_13095 and JI728_13100) or membrane proteins (*nhaA*) to counteract the damage (37, 38). These findings indicated that the selected genes were highly correlated with the resistance phenotype and were essential for the survival of *S*. Pullorum R51 in egg albumen at different temperatures.

### Downregulated gene further prioritizes bacterial metabolism

**Fig 4:**
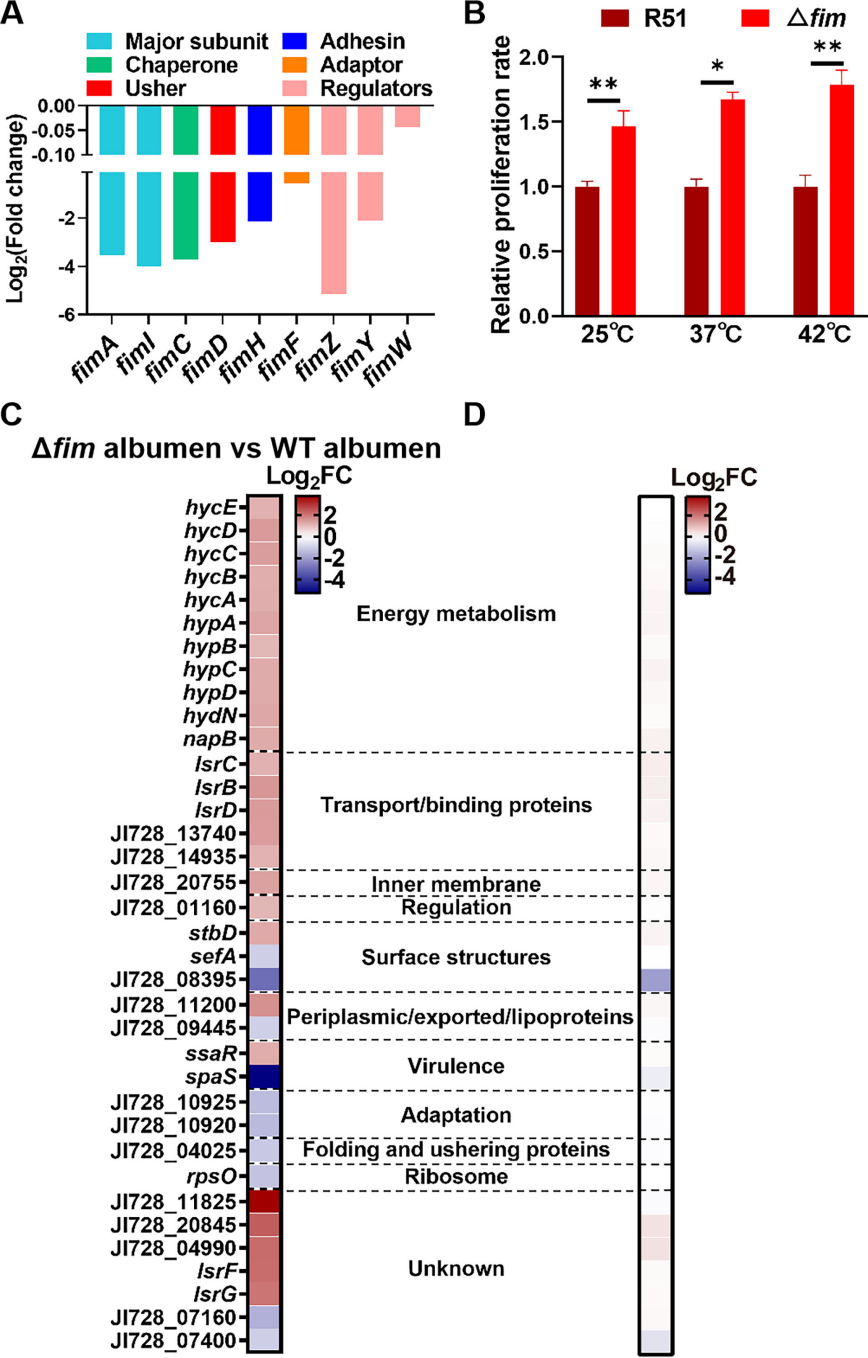
Downregulated gene further prioritizes bacterial metabolism. (A) The genes within the *fim* cluster were downregulated in the albumen compared to LB. (**B**) The *fim* deletion increased the proliferation in the albumen under three temperatures. (**C**) The function classification of 36 DEGs of the Δ*fim* mutant in the albumen compared to the wild type in the egg albumen. (**D**) The Log_2_(fold change) of the above 36 DEGs of the Δ*fim* mutant compared to the wild type in LB broth. The unpaired *t*-test was used to analyze the differences between wild type and Δ*fim* mutant. *P < 0.05 and **P < 0.01.

In our transcriptome analysis, we observed a significant downregulation of all genes within the *fim* gene cluster (Fig. 4A), different from the other five fimbrial appendages in *S*. Pullorum (Table S2). Previous studies have reported a significant decrease in *fimA* expression in *S*. Enteritidis following exposure to egg white (13). In our previous work with low-dose *S*. Pullorum inoculation, the *fimD* deletion mutant exhibited enhanced survival capability compared to the wild-type strain (18). To examine whether the Δ*fim* mutant displays a comparable phenotype at high doses, the wild-type and *fimD* deletion strains were inoculated into egg white at a final concentration of 5 × 10^7^ CFU/mL, respectively. The results showed a significant survival advantage for the deletion strain at three temperatures, underscoring the role of the downregulated gene *fimD* (Fig. 4B). To further understand the underlying reason, we further investigated the expression profile of the *fim* deletion strain in egg white by RNA-Seq analysis. The results revealed that egg albumen exposure induced 26 differentially upregulated genes and 10 differentially downregulated genes compared to wild type (Fig. 4C). Classifying the 36 DEGs into functional categories (19, 39), the majority were associated with energy metabolism (11 genes), transporting or binding protein (5 genes), which exhibited significantly upregulated expression levels (39) (Fig. 4C; Table S5). Importantly, among the metabolism-related genes, *hycABCDE*, *hypABCDE,* and *hydN* were involved in hydrogen production and utilization. We speculated that the Δ*fim* mutant, unable to attach to egg albumen surfaces, switched to growth by fermentation due to low oxygen. This fermentation process resulted in the production of formic acid, which was further metabolized to H_2_ by the formate hydrogenlyase (FHL) system. The production of hydrogen could potentially contribute to proton gradients across membranes, thereby conserving energy (40, 41). These genes in the Δ*fim* mutant under LB broth showed no significant changes compared to the wild-type strain (Fig. 4D; Table S6), consistent with the previously observed similar growth rates of both strains under LB conditions (18).

## DISCUSSION

*Salmonella* Pullorum is an avian-restricted biovar, one of serovar *Salmonella* Gallinarum, causing pullorum disease in newly hatched birds (42, 43). Vertical transmission is considered an efficient mode of dissemination due to its ability to transmit from infected hens to offspring (18, 26). During this process, completing egg (embryo) transmission is an indispensable part. Despite eggs developing from various parts of the reproductive system, not all pathogens that can colonize these organs have the ability to transmit vertically. For example, *S*. Typhimurium can be detected in the reproductive organs but not inside the formed eggs (34). This emphasizes surviving and persisting within the eggs for *S*. Pullorum to achieve vertical transmission. Egg white is a hostile environment with numerous antibacterial substances. Despite this, *S*. Pullorum can persist in the egg white of infected hens for up to 4 weeks, with a bacterial load exceeding 10^3^ CFU/g (26). Very little is known, however, about the survival mechanisms of S. Pullorum in the context of egg albumen.

**Fig 5:**
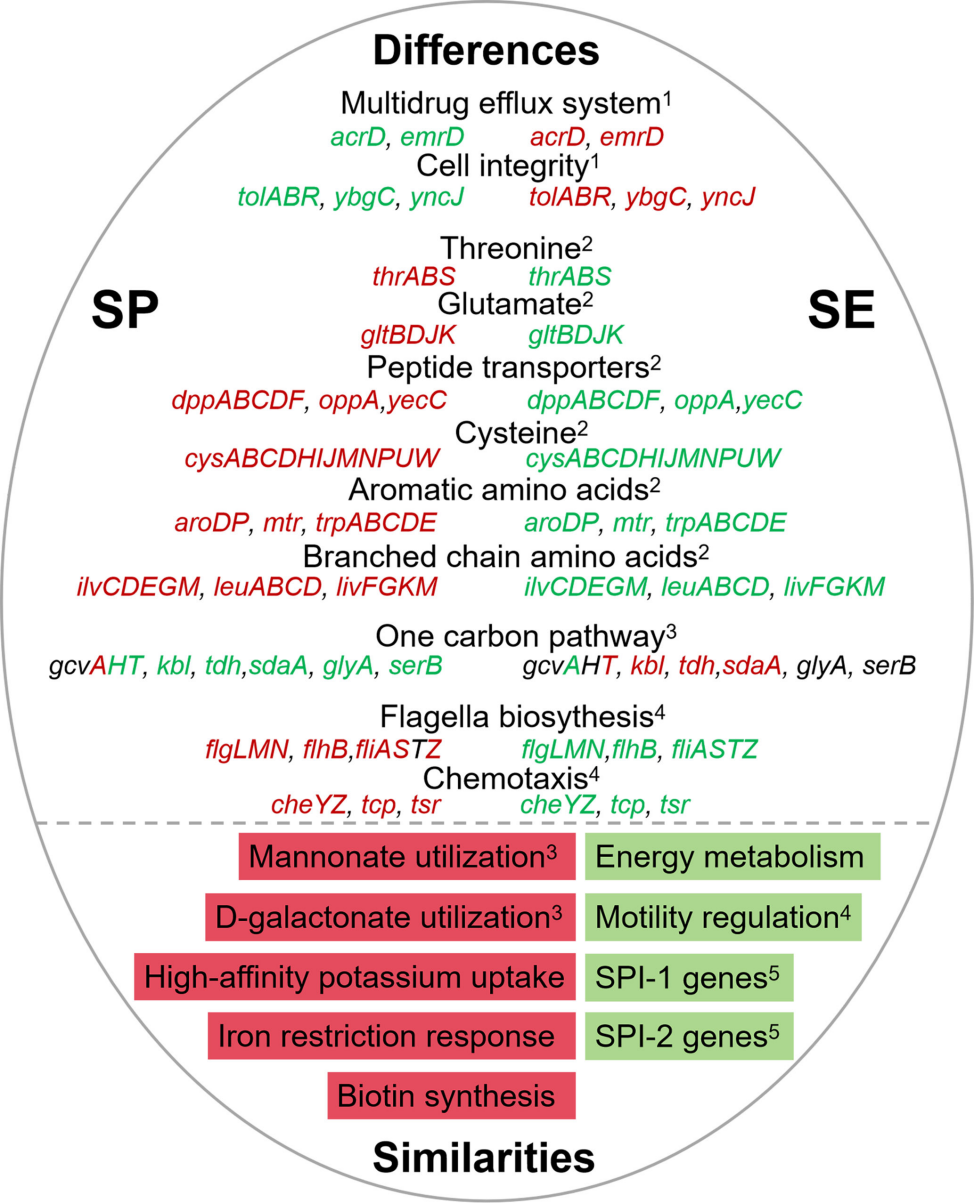
Comparative analysis of genes and pathways between *S*. Pullorum (SP, in this study) and *S*. Enteritidis (SE) after exposure to the egg white (13, 28). The upper and lower sections show the differences (SP on the left and SE on the right) and similarities, respectively. Red font or filling represents upregulated genes, while green indicates downregulated genes. The numbers in the top right corner of the functional category indicate their classification within major category groups. 1, membrane-stress related genes; 2, metabolism and transport of amino acids; 3, hexonate and hexuronate utilization and carbohydrate metabolism; 4, motility; and 5, virulence.

In the current study, we employed transcriptome sequencing analysis to investigate the global gene profile of *S*. Pullorum during the early stage (6 h) of egg white formation. Among the 1,606 differentially expressed genes (Fig. 1B), many exhibited expression patterns consistent with *S*. Enteritidis in previous studies (13, 28). For example, the iron-starvation and manganese uptake genes showed upregulated expression, while virulence genes and energy metabolism-related genes, such as those within *hyc* and *nuo* operons responsible for formate hydrogenlyase and NADH dehydrogenase, respectively, were significantly downregulated (Fig. 5; Table S7) (13, 28). However, we observed that many genes exhibited entirely distinct expression profiles between the two serovars, particularly those associated with amino acid metabolism and transport, in which the expressions were significantly upregulated in *S*. Pullorum but downregulated in *S*. Enteritidis (Fig. 5). For example, three *cys* gene clusters related to sulfate assimilation and cysteine biosynthesis (*cysDNC*, *cysJIH*, and *cysPTWAM*) (44) and genes within *ilv*, *leu*, and *liv* clusters related to branched-chain amino acids’ biosynthesis (iso-leucine, leucine, and valine) showed a significant upregulation, contrary to findings observed by Baron et al. (28). One plausible explanation of these findings is that *S*. Pullorum, in contrast to *S*. Enteritidis, lacks seven fimbrial appendages including *pef*, *sef*, and *stb*. This deficiency might diminish the ability to adhere to the surface of egg white, thereby compelling *S*. Pullorum to utilize more amino acids and other nutrients for survival (19, 45).

Interestingly, *S*. Pullorum is generally considered aflagellate and nonmotile, with flagella production induced only in specialized culture media like Hektoen enteric (HE) medium supplemented with iron (46). Our results found that some genes related to motility, like *fliLMN*, *fliASZ,* and *cheYZ*, were upregulated. We assumed that the upregulated iron synthesis-related genes in egg white increased iron levels and mimicked the HE medium to some extent. Moreover, our results uncovered that the long *eut* operon with 17 genes responsible for ethanolamine utilization was downregulated, which has not been mentioned elsewhere. We speculated that *S*. Pullorum utilized other carbon sources like glucose in the albumen rather than endogenous ethanolamine (47).

Among numerous DEGs, we innovatively integrated comparative genome analysis to pinpoint potential candidates contributing to the vertical transmission of *S*. Pullorum (Fig. 2A), which was based on the distinction that *S*. Pullorum and *S*. Enteritidis can transmit vertically while *S*. Typhimurium lacks the ability. Survival assays in egg white revealed that upregulated functionally relevant genes play a significant role. We speculated that these genes reprogram the metabolism profile to conserve energy since half of the top 10 upregulated KEGG enrichment pathways were related to metabolic pathways (Fig. 1D). Nevertheless, comparative genomics usually missed genes on plasmids. R51 contains two plasmids, CP068387.1 and CP068388.1, with 27 and 25 significantly upregulated genes, respectively, which may play a potential role during vertical transmission. Besides, even though the antibacterial components and pH between fertilized and unfertilized eggs during the early stage were similar (48, 49), the significant difference was ovalbumin, comprising about 55% of the total proteins in egg albumen, which functions to store the proteins (50). We cannot neglect continuous monitoring in the dynamic process of chicken embryo incubation since egg white weight gradually decreases, accompanied by changes in its composition (49, 51), which might cause a distinct response in *Salmonella*.

*Salmonella* type 1 fimbriae are adhesive structures on the surface of the bacteria, facilitating their attachment to host cells and surfaces (52). Its deletion mutant was more frequently isolated in the oviduct after infecting hens (53), where egg albumen forms. The promoter *fimA* in the *fim* cluster of *S*. Enteritidis was induced in egg white at 42°C in promoter trap technology by Raspoet et al. (54). Conversely, a study using RNA sequencing analysis conducted by Huang et al. (13) revealed that all the genes within *fim* operon were significantly downregulated at 6, 12, and 24 h after *S*. Enteritidis was exposed to egg albumen. Another study inoculating *S*. Enteritidis in egg white for 7, 25, and 45 min showed similar results (28), likely due to different experimental methods, with the high resolution and high-throughput advantages of RNA-Seq (55). Our results revealed identical downregulated expression (Fig. 4A), further validated by survival ability comparisons in egg white between wild type and Δ*fim* mutant (Fig. 4B).

Previously, few studies focused on downregulated genes. In the current study, there were 844 downregulated DEGs, outnumbering the upregulated 762 genes. Among the top 10 enriched downregulated KEGG pathways, the pathway related to bacterial invasion of epithelial cells had the highest score (Fig. 1D). These compelled us to investigate further the role of fimbriae in the survival of *S*. Pullorum in egg white. Compared with wild type in the egg white, the Δ*fim* mutant exhibited 36 DEGs, with 26 upregulated genes, almost half of which were related to energy metabolism within *hyc* and *hyp* operon (Fig. 4C). Hyc genes encoding the hydrogenase-3 complex required for the formation of the FHL system and *hyp* genes required for the post-translational formation of the hydrogenases were regulated by the transcriptional activator FhlA (56). The product of *hydN* is proposed to contribute to electron flow from or to formate dehydrogenase H (57, 58). NapB, a small nitrate reductase cytochrome c-type subunit, prevents concurrent nitrate and nitrite respiration and contributes to anaerobic respiration (59, 60). These indicated that under static conditions with insufficient oxygen supply, the absence of *fim* greatly enhanced the activity of formate hydrogenlyase-related genes to release hydrogen and conserve energy, thereby sustaining its survival and growth (61). In addition, *lsr* operon plays a role in autoinducer-2 (AI-2) internalization and ATP hydrolysis during transport (62, 63). The product of gene JI728_14935 functions as an ATP-binding cassette domain-containing protein. AI-2 acting as a signal molecule of quorum sensing along with consumption of ATP has been identified to regulate various bacterial behaviors, such as survival and biofilm formation (62). Therefore, the increased expression of the Lsr genes and JI728_14935 resulting from *fim* deletion might lead to enhanced survival ability in *S*. Pullorum. Moreover, the gene JI728_13740 (encoding MFS transporter) showed higher expression levels in the Δ*fim* mutant, facilitating increased absorption of nutrients and protecting against harmful substances (42). We speculated that *fim* might somehow suppress the expression of these genes, which will be the focus of our future work.

### Conclusion

In summary, this study used RNA-Seq and comparative genomic analysis, termed integrated OMICs, to investigate functionally relevant genes for *Salmonella* survival in egg white, focusing on both up- and downregulated genes. Initial RNA-Seq analysis showed dramatic gene profile changes within egg white compared with the LB condition. Considering that *S*. Pullorum and *S*. Enteritidis are recognized agents for vertical transmission and survival in egg white, while *S*. Typhimurium cannot, further comparative genomic analysis narrowed down 136 genes unique to *S*. Pullorum and *S*. Enteritidis. Five functionally relevant gene mutants were confirmed by egg white survival assays, with genes JI728_13095 and JI728_13100 closely associated with the energy pathway. Among the downregulated genes, we found that a *fim* operon mutant could promote *S*. Pullorum in egg white. Second-round RNA-Seq data of the Δ*fim* mutant in egg white showed several significantly upregulated genes pointing to energy metabolism-related genes within *hyc* and *hyp* gene clusters, consistent with the initial RNA-Seq results. The collective findings indicated that energy metabolism could promote *Salmonella* Pullorum persistence in egg white, guiding new interventions in future studies.

## MATERIALS AND METHODS

### Bacterial strains and plasmids

Bacterial strains, plasmids, and primers used in this study are listed in Tables S8 and S9, respectively. A clinical isolate of *S*. Pullorum R51, which exhibits strong survival ability in albumen (18, 64), was used. All strains were generally cultured in LB medium (Oxoid, Thermo Scientific, USA) and incubated at 37°C. Strains with the temperature-sensitive plasmid pCas were grown at 30°C. Kanamycin (50 µg/mL) or spectinomycin (100 µg/mL) were added when needed.

### Egg albumen preparation

The egg albumen was prepared as previously described (43), with minor changes. Fresh specific-pathogen-free eggs were purchased from Ningbo Chunpai Agriculture Technology (Zhejiang, China). The egg surfaces were sanitized with 75% ethanol, and the albumen was separated and placed into a large sterilized container. After thoroughly stirring without clumped colloid, the mixture was centrifuged twice at 12,000 × *g* for 5 min. The supernatant was transferred to a sterile bottle and stored at 4°C for up to 4 days.

### Incubation of strains in egg white

To choose a proper incubation dose, single colonies of *S*. Pullorum strain R51 and *E. coli* strain DH5α were grown overnight in LB broth at 37°C with shaking. After centrifugation at 4,000 × *g* for 10 min, the cells were washed thrice, resuspended in 1× PBS, and the optical density at 620 nm was adjusted to 0.5 (1 × 10^9^ CFU/mL). To achieve the same final albumen concentration, the resulting suspension was diluted to 5 × 10^8^, 2.5 × 10^8^, and 5 × 10^7^ CFU/mL, 400 µL of which was added to 1.6 mL of prepared egg white, respectively. The inoculated media were incubated at 42°C for 6 h to evaluate their antibacterial activities.

For the RNA-Seq sample preparation, the bacterial culture and albumen inoculation were performed as described above. A volume of 50 mL bacterial suspension with a final concentration of 5 × 10^7^ CFU/mL was placed into 250-mL conical flasks and incubated at 42°C for 6 h. Meanwhile, the same volume and concentration suspensions were incubated in LB broth as a control. The culture was centrifuged at 12,000 × *g* at 4°C, the supernatant was removed, and the tubes were inverted onto paper to remove the liquid as much as possible (13). The pellets were immediately frozen in liquid nitrogen and stored at −80°C for later RNA extraction. Three independent biological replicates were prepared for each condition.

To assess the survival abilities between wild-type and mutant strains in egg albumen, the same method was utilized, adding two temperature conditions, 25°C and 37°C. The former resembles the natural egg storage condition, while the latter mimics the temperature during egg incubation. The relative proliferation rate was calculated as the proliferation rate of mutant strains/the proliferation rate of wild-type R51. The proliferation rate of each strain was calculated as the final bacterial concentration after incubation/initial inoculum concentration. Three independent biological replicates were prepared for each strain and each time point.

### RNA sequencing and data analysis

The total RNA of all strains was extracted with Trizol reagent (Invitrogen, USA) and assessed using Thermo NanoDrop One and Agilent 4200 Tape Station instruments. Then, the rRNA was eliminated using the Epicentre Ribo-Zero rRNA Removal Kit. Subsequently, the NEBNext Ultra II Directional RNA Library Prep Kit for Illumina was used for cDNA library construction. This process was completed by Magigene Biotechnology (Guangdong, China).

Clean data were obtained by trimming the sequencing adaptors, removing the reads with low quality, and mapping them against the reference genome of the R51 strain (GenBank accession number CP068386.1) and two plasmids (CP068387.1 and CP068388.1) with Bowtie 2 (65). The fragments per kilobase per million reads value was used to eliminate the effect of gene length and sequencing depth. DEGs were calculated using DESeq2 (66), identified as those with FDR ≤ 0.05 and |log_2_(fold change) | ≥1, and were analyzed against the Gene Ontology and KEGG database to complete biological function and pathway annotation.

### Real-time quantitative PCR assays

To verify the RNA sequencing data, seven genes with significant expression were selected to perform RT-qPCR assays (18). Total RNA was extracted using the RNAprep Pure Cell/Bacteria Kit (Tiangen, Beijing, China). Residual genomic DNA removal and cDNA synthesis were conducted using HiScript III RT SuperMix for qPCR (+gDNA wiper) (Vazyme Biotech Co., Ltd., Nanjing, China) following the manufacturer’s instructions. The PCR reactions were then performed in triplicate using the Taq Pro Universal SYBR qPCR Master Mix (Vazyme Biotech Co., Ltd., Nanjing, China). The 2^-ΔΔCt^ cycle threshold (Ct) method was used to analyze the relative transcriptional levels of target genes, and *gapA* was used as the internal control gene. All experimental results were performed in three biological replicates.

### Comparative genomic analysis

We employed a web-based tool, PanExplorer, to perform the comparative genome analysis (http://panexplorer.southgreen.fr) (35). The program PanACoTA was chosen to conduct the pan-genome (67). The four GenBank assembly accession numbers (R51, GCA_027474565.1; P125109, GCA_000009505.1; LT2, GCA_014334155.1; and SL1344, GCA_000210855.2) were entered. The minimum percentage identity for BLAST was set to 95%. The five genes were confirmed exclusive to R51 and P125109 by manual alignment.

### Construction of targeted mutants

The targeted deletion mutants were constructed by homologous recombination via the CRISPR/Cas9-mediated genome-editing system (18, 43, 68, 69), with minor changes. The sgRNA was designed through the online web server CHOPCHOP (http://chopchop.cbu.uib.no) and amplified with the primers gene-sgRNA-F/sgRNA-R. The 500-bp upstream and downstream fragments (Xf and Xr) of the target region were amplified with two pairs of primers (gene-Xf-F/gene-Xf-R and gene-Xr-F/gene-Xr-R). The sgRNA, Xf, and Xr fragments were purified by the FastPure Gel DNA Extraction Mini Kit (Vazyme Biotech Co., Ltd., Nanjing, China) and homologously recombined into plasmid pTargetF by the Uniclone One Step Seamless Cloning Kit (Beijing Genesand Biotech Co., Ltd) to obtain plasmid pTargetT. The pTargetT was extracted by the Easy Plasmid Miniprep Kit (Zhejiang Easy-Do Biotech Co., Ltd) and chemically transformed into R51 competent cells harboring temperature-sensitive plasmid pCas to generate mutant strains followed by PCR identification and sequencing. Finally, the plasmid pTargetT was cured by isopro-pyl-D-thiogalactopyranoside (0.5 mM), and plasmid pCas was cured by incubation at 42°C for 3 days.

### Statistical analysis

The results were analyzed using GraphPad Prism 9.0.0 (San Diego, CA, USA). The means ± standard error of the mean was determined based on three independent experiments. The unpaired *t*-test and one-way analysis of variance test were used to analyze the differences. A value of *P* < 0.05 was considered statistically significant.

## Data Availability

The RNA-Seq data have been deposited in the Gene Expression Omnibus (GEO) database under accession no. GSE256211.

## SUPPLEMENTAL MATERIAL

Supplemental tables msphere.00362-24-s0001.xlsx Tables S1 to S9.

ASM does not own the copyrights to Supplemental Material that may be linked to, or accessed through, an article. The authors have granted ASM a non-exclusive, world-wide license to publish the Supplemental Material files. Please contact the corresponding author directly for reuse.
